# An Analysis of the Top-cited Articles in Emergency Medicine Education Literature

**DOI:** 10.5811/westjem.2016.10.31492

**Published:** 2016-11-23

**Authors:** Brendan W. Munzer, Jeffery Love, Barbara L. Shipman, Brendan Byrne, Stephen J. Cico, Robert Furlong, Sorabh Khandelwal, Sally A. Santen

**Affiliations:** * University of Michigan, Department of Emergency Medicine, Ann Arbor, Michigan; ‡ University of Michigan, Alfred Taubman Health Sciences Library, Ann Arbor, Michigan; † Georgetown University Hospital/Washington Hospital Center, Department of Emergency Medicine, Washington, D.C.; § Naval Medical Center Portsmouth, Department of Emergency Medicine, Portsmouth, Virginia; ¶ Indiana University, Department of Emergency Medicine, Indianapolis, Indiana; || Ohio State University, Department of Emergency Medicine, Columbus, Ohio; # University of Michigan, Department of Learning Health Sciences, Ann Arbor, Michigan

## Abstract

**Introduction:**

Dissemination of educational research is critical to improving medical education, promotion of faculty and ultimately patient care. The objective of this study was to identify the top 25 cited education articles in the emergency medicine (EM) literature and the top 25 cited EM education articles in all journals, as well as report on the characteristics of the articles.

**Methods:**

Two searches were conducted in the Web of Science in June 2016 using a list of education-related search terms. We searched 19 EM journals for education articles as well as all other literature for EM education-related articles. Articles identified were reviewed for citation count, article type, journal, authors, and publication year.

**Results:**

With regards to EM journals, the greatest number of articles were classified as articles/reviews, followed by research articles on topics such as deliberate practice (cited 266 times) and cognitive errors (cited 201 times). In contrast in the non-EM journals, research articles were predominant. Both searches found several simulation and ultrasound articles to be included. The most common EM journal was *Academic Emergency Medicine* (n = 18), and *Academic Medicine* was the most common non-EM journal (n=5). A reasonable number of articles included external funding sources (6 EM articles and 13 non-EM articles.)

**Conclusion:**

This study identified the most frequently cited medical education articles in the field of EM education, published in EM journals as well as all other journals indexed in Web of Science. The results identify impactful articles to medical education, providing a resource to educators while identifying trends that may be used to guide EM educational research and publishing efforts.

## INTRODUCTION

Dissemination of educational research evidence is critical to improving medical education and ultimately patient care. One reasonable measure of the impact of a publication is the number of citations a particular work receives. This number is indicative of the dissemination of its results and serves as a measure of the work’s service as a foundation for supporting further research.

Publications and the number of citations also serve as important criteria on which faculty are promoted at some institutions, though other institutions do not use number of citations as a promotional criterion. The number of citations does not solely reflect the work of an individual or team, but also serves as a metric for evaluating the research performed at a departmental, institutional, or even national level.^1^ The number of citations of publications may provide one measure by which to determine the impact of work. Additionally, other factors, such as funding, are often used to assess productivity in the research community.

Examination of citations is rarely performed except for the purpose of putting together a promotion package. Azer performed bibliometric analyses evaluating the top-cited articles in medical education;^2^ however, there is currently no literature describing the top-cited education articles within the field of emergency medicine (EM). This type of intentional examination can have a number of benefits. First, when examining which articles have received the most citations, it becomes possible for researchers to more easily familiarize themselves with the landmark articles within a field. Second, it provides researchers with information on which topic areas, journals, and research methods tend to be more highly cited. This indicates not only the quality of the research but also the translational impact of the work.^3^ Third, as funding for medical education research is difficult, by evaluating the funding sources of highly cited articles, this type of examination allows researchers to identify sources of potential funding.

The objective of this study was to identify the top 25 cited education articles in the EM literature and report on their characteristics, as well as the top 25 cited EM education articles in all other indexed journals. We sought to provide clinicians, educators, and researchers with resources for identifying the highest-impact literature in emergency medical education and a database of options to explore when looking to publish within the field of medical education.

## METHODS

Within two distinct searches in the Web of Science index, we identified the top 25 articles related to education and EM. The EM journal search was limited to 19 EM journals (Appendix 1). EM-related journals eligible for inclusion were English-language journals indexed within PubMed. Exclusion criteria for these journals included non-English language journals, prehospital journals, and journals with a non-physician focus. We ran the second search within all other indexed journals, excluding the EM journals that were excluded in the first search in Web of Science.

Inclusion criteria for each individual article were the following: it had to be primarily EM-related or include emergency physician subjects and be relevant to EM education; its content was deemed educational; it had to be published in English and it had to have subjects that included physicians or future physicians. Among the exclusion criteria for individual articles were these: the subjects did not include EM residents or physicians or medical students in an emergency department setting; or research subjects were non-hospital based (such as EMS or community-based teaching).

From May 18-June 2, 2016, the authors used keywords and search tools within the Web of Science database to retrieve the top-cited articles in both categories. The aim of this search was to identify not only the most highly cited education articles published in EM journals but also the most highly cited EM-based articles related to education that been published in other literature. The keywords were for the large part those used by Azer.^2^ (See Appendix 1 for search strategy)

Articles were placed in descending order of number of citations in an Excel spreadsheet. Two of the authors then independently assessed both search categories and applied inclusion and exclusion criteria Inter-rater reliability among assessors for selection of the top-cited EM articles, calculated using Cohen’s kappa, was acceptable (0.69). The top 25 cited articles involving EM and education were identified and placed into a final list (“Top Cited Education Articles in EM Literature” and “Top Cited EM-related Education Articles”).

Articles were assigned for review and divided evenly between the author group. Two authors independently reviewed the full text for each article and recorded the following information: (1) article name; (2) first author; (3) source journal; (4) year of publication; (5) number of citations; funding source (if applicable); (6) journal impact factor; (7) journal discipline; (8) article type; (9) educational content; (10) subjects; and (11) research method (if applicable). Each author-pair discussed the outcomes of this data collection to create a consensus. If any discrepancies arose, a third author evaluated the article in question and provided a tiebreaker. Finally, findings were discussed in conference with all authors.

## RESULTS

Table 1 summarizes the 25 top-cited medical education articles in EM journals.^4^–^28^ Table 2 summarizes the 25 top-cited medical education articles involving EM in all other journals.^29^–^52^ Articles are listed in descending order with a rank from 1–25 based upon the number of citations, as found in Web of Science at the time of the search.

With regards to EM journals, the greatest number of articles were classified as articles/reviews. The most frequently cited article was “Deliberate Practice and Acquisition of Expert Performance: A General Overview” by Ericsson, published in *Academic Emergency Medicine* in 2008 and based on a consensus preconference.^12^ It had been cited 266 times. Six articles were research papers and seven were curriculum, four of which included a research methodology. One article was a needs assessment. The topics included simulation; learning theory; ultrasound; assessment; learner retention; and interprofessional education. The top three most-cited articles all exhibited a focus on learning theory. Table 3 summarizes these results.

Articles were published most commonly in *Academic Emergency Medicine* (n = 18; 72%), *Annals of Emergency Medicine* (n = 4; 16%); and *Resuscitation* (n = 2; 8%). The majority of articles (n = 19; 76%) listed no funding, while five articles (20%) received external funding alone. One article received both internal and external funding.

With regards to the articles in other journals, the majority were research papers. There were seven curriculum articles and two articles/reviews. The topics included simulation; professionalism; management practice; ultrasound; assessment; cross-cultural care; error in diagnosis; learner retention; specialty choice; and supervision.

Articles in the second literature search were from a wide variety of journals including *Academic Medicine* (n = 5; 20%); *Medical Education* (n = 3; 12%); *British Medical Journal* (n = 2; 8%)*; Journal of the American Medical Association*; and *Pediatrics* (n = 2; 8%). The most frequently cited article was “Prospective Analysis of a Rapid Trauma Ultrasound Examination Performed by Emergency Physicians” by Ma, published in *Journal of Trauma* in 1995 and cited 193 times. Eight articles had no funding (32%), 13 (52%) were funded by external awards, one article (4%) was funded solely through internal grants, while three (12%) received both internal and external funding. Table 4 summarizes these results.

We calculated the Pearson correlation coefficient (*r*) to determine if the age of the article was correlated to the number of citations received. For the articles in the non-EM journals, there was a negative correlation between the year of publication and the number of citations (r = 0.42), meaning that the more recently published articles were cited less often. For the EM journals, however, this correlation was not seen (r = 0.2). Articles from Table 2 (Non-EM) published in higher impact journals were cited more often (r = 0.46). This was not the case for articles in the EM journal search (r = 0.03).

## DISCUSSION

This study identified the top 25 most frequently cited EM education articles in both EM literature as well as the remainder of journals based in the Web of Science index. The findings of this study provide information regarding pertinent trends and topics in EM education, as noted in Table 3 and Table 4, while providing an accessible location to identify some of the highest-impact literature within this field. Additionally, it allows us to take note of the journals in which EM education is most often recognized and published, serving as an historical perspective for those seeking to publish work.

It is apparent that there are trends both with regards to the overall field of EM education, as well as the journals in which these articles are published. Non-EM journals have, on average, a higher impact factor (up to 35 for *JAMA*), indicating that they have a higher number of cited articles and therefore are likely distributed to a wider audience. It then makes sense that, when appropriate, authors would seek to submit articles to a wider-reaching journal. For instance, the largest number of highly cited articles in both groups of journals was simulation. This suggests that simulation is a topic that has both specialty-specific and wide-reaching interest. The top two cited articles focused on simulation located in non-EM journals (Shapiro et al. and Barsuk et al.) are both more highly cited than the top ranked simulation article in EM journals. An author looking to publish an article involving simulation would therefore need to balance the benefits of publishing within the field of EM, with associated peer recognition, against the benefits of publishing in a journal with higher impact. It is interesting to note that the top two cited articles in this study are located in EM journals. This confounds the idea that a wider audience will provide a greater number of citations overall and is possibly related to specialty association and peer recognition.

Table 1 and Table 2 provide information regarding the type of article, with the finding that the vast majority of articles in the non-EM journals were found to be research articles, while EM journals tended to have more articles/reviews that were highly cited. This suggests that there is a preference in publication for research-driven articles in non-EM journals. In contrast, Ericsson’s theory-based article was significant in EM, as was Croskerrv’s article indicating that publishing a key learning theory paper in EM may also provide a meaningful foundation.^11^, ^12^

The most common EM journal in which highly cited medical education articles were published was *Academic Emergency Medicine*. Medical education articles previously considered for publication in *Academic Emergency Medicine* will now be directed to submit to the new journal *Academic Emergency Medicine Education and Training*. It should be noted that because this journal will not be indexed for several years, articles published in this journal would not have been considered for this ranking list.

Within non-EM journals, roughly half were specialty focused (i.e., *Pediatrics*, *Journal of Trauma: Injury, Infection, and Critical Care*) and half were general medical journals. Articles included within specialty journals tended to have a focus that was less specific to the specialty of EM. It was of note that some of these articles chose a generalizable topic such as simulation or depression and used a subject population that included EM residents as well as other specialties.

The articles published within non-EM journals had a larger number of authors who received funding, whether internal or external funding, than articles published in EM journals. It should be noted, however, that the top-cited articles in both the EM journals and the non-EM journals did not have any funding. While it can be helpful to have the support that funding provides, this finding suggests that unfunded work is worthwhile and can still be impactful.

One goal of education research is to disseminate educational practices.^3^ Many articles have been widely disseminated despite not being highly cited. For instance, all EM residencies use the “Standardized Letter of Evaluation” (SLOE), as one way of reviewing potential applicants; however, the paper describing its predecessor, the “Standardized Letter of Recommendation (SLOR) and subsequent SLOE papers would not appear on the top 25 cited list in this article.^53^, ^54^ This suggests that citation numbers alone do not provide all information regarding the reach of research being performed.

This study provides a repository for some of the most impactful literature in EM medical education. For example, the articles on deliberate practice and cognitive strategies for de-biasing are important foundations for EM education. Additionally, some research articles form the basis for further research and educational development. By collecting these articles in one location, it allows others to discover landmark articles within the field of medical education. It also allows others to identify trends in EM education research, note common funding sources, and advance the field of medical education.

## LIMITATIONS

Limitations of this study include the fact that articles were searched in only one database, the Web of Science. It is possible that a search performed in a different database, such as SCOPUS, may have provided additional articles or slightly different search findings. For instance, the EM journals were identified a priori of the search. The *Journal of Trauma – Injury Infection and Critical Care* was not originally identified in that list but could be considered an EM journal. We chose to leave it in the non-EM list based on the a priori listing of journals. Additionally, we excluded articles and journals if they were not English language. This may have skewed search results to favor a Western viewpoint while neglecting articles that may have had additional global influence. Another limitation was our attempt to define what constitutes education research as related to education and training. This may have added a measure of subjectivity, although our inter-rater kappa was acceptable. One final limitation is that this article did not identify where articles were cited, and the subsequent reach of these articles, as well as self-citations.

As related above, the ability to determine impact based upon citation count alone is difficult as there are widely read articles that are not highly cited. Citation counts do provide a foundation; further research could identify what qualities make an article more likely to be disseminated.

## CONCLUSION

This study identified the most frequently cited medical education journals in the field of emergency medicine, published in EM journals as well as all other journals indexed in Web of Science. The results identify impactful articles that are collected in one location, providing a resource to others while identifying trends that may be used to guide emergency medicine educational research and publishing efforts.

## Supplementary Information



## Figures and Tables

**Table 1:** Most cited education articles from emergency medicine journals.

Rank	First author; year	Title	Journal: impact factor	Category	Funding (if present)	Number of citations
1	Ericsson, KA; 2008	Deliberate Practice and Acquisition of Expert Performance: A General Overview	*Academic Emergency Medicine*; 2.0	Article/Review		266
2	Croskerry, P; 2002	Achieving Quality in Clinical Decision Making: Cognitive Strategies & Detection of Bias	*Academic Emergency Medicine*; 2.0	Article/Review	Combined (AHQR Grant)	201
3	Croskerry, P; 2003	Cognitive Forcing Strategies in Clinical Decisionmaking	*Annals of Emergency Medicine*; 4.7	Article/Review	External (AHQR Grant)	132
4	Mateer, J; 1994	Model Curriculum for Physician Training in Emergency Ultrasonography	*Annals of Emergency Medicine*; 4.7	Curriculum - No Data		127
5	Small, SD; 1999	Demonstration of High-fidelity Simulation Team Training for Emergency Medicine	*Academic Emergency Medicine*; 2.0	Curriculum - No Data	External (Medsim-Eagle Simulation, Inc./Army Research Laboratory)	123
6	Rudolph, JW; 2008	Debriefing as Formative Assessment: Closing Performance Gaps in Medical Education	*Academic Emergency Medicine*; 2.0	Article/Review		100
7	Vozenilek, J; 2004	See one, Do one, Teach one: Advanced Technology in Medical Education	*Academic Emergency Medicine*; 2.0	Article/Review		89
8	Reznek, M; 2003	Emergency Medicine Crisis Resource Management: Pilot Study of a Simulation-based Crisis Management Course for Emergency Medicine	*Academic Emergency Medicine*; 2.0	Curriculum - Yes Data		86
9	Swing, SR; 2002	Assessing the ACGME General Competencies: General Considerations and Assessment Methods	*Academic Emergency Medicine*; 2.0	Article/Review	External (Robert Wood Johnson Foundation)	86
10	Campbell, JC; 2001	An Evaluation of a System-change Training Model to Improve Emergency Department Response to Battered Women	*Academic Emergency Medicine*; 2.0	Curriculum - Yes Data	External (Centers for Disease Control)	81
11	Perkins, GD; 2007	Simulation in Resuscitation Training	*Resuscitation*; 4.2	Article/Review	External (DH [NIHR] Clinician Scientist Award)	73
12	Mower, WR; 1999	Evaluating Bias and Variability in Diagnostic Test Reports	*Annals of Emergency Medicine*; 4.7	Article/Review		67
13	McLaughlin, SA; 2002	Human Simulation in Emergency Medicine Training: A Model Curriculum	*Academic Emergency Medicine*; 2.0	Curriculum - No Data		64
14	Mandavia, DP; 2000	Ultrasound Training for Emergency Physicians - A Prospective Study	*Academic Emergency Medicine*; 2.0	Curriculum-Yes data		63
15	Kuhn, GJ; 2002	Diagnostic Errors	*Academic Emergency Medicine*; 2.0	Article/Review		62
16	Cooper, S; 2010	Rating Medical Emergency Teamwork Performance: Development of the Team Emergency Assessment Measure (TEAM)	*Resuscitation*; 4.2	Research		57
17	Bond, WF; 2007	The Use of Simulation in Emergency Medicine: A Research Agenda	*Academic Emergency Medicine*; 2.0	Needs Assessment		56
18	Blaivas, M; 2003	Short-axis Versus Long-axis Approaches for Teaching Ultrasound-guided Vascular Access on a New Inanimate Model	*Academic Emergency Medicine*; 2.0	Research		56
19	Jabbour, M; 1996	Life Support Courses: Are They Effective?	*Annals of Emergency Medicine*; 4.7	Article/Review		54
20	Jones, AE; 2003	Focused Training of Emergency Medicine Residents in Goal-Directed Echocardiography: A Prospective Study	*Academic Emergency Medicine*; 2.0	Research		52
21	Counselman, FL; 2003	The Status of Bedside Ultrasonography Training in Emergency Medicine Residency Programs	*Academic Emergency Medicine*; 2.0	Curriculum-Yes data		50
22	Kovacs, G; 1999	Clinical Decision Making: An Emergency Medicine Perspective	*Academic Emergency Medicine*; 2.0	Article/Review		50
23	Burdick, WP; 1995	Observation of Emergency-Medicine Residents at the Bedside - How often Does It Happen?	*Academic Emergency Medicine*; 2.0	Research		50
24	Gisondi, MA; 2004	Assessment of Resident Professionalism using High-fidelity Simulation of Ethical Dilemmas	*Academic Emergency Medicine*; 2.0	Research		48
25	Santora, TA; 1996	Video Assessment of Trauma Response: Adherence to ATLS Protocols	*American Journal of Emergency Medicine*; 1.3	Research		48

*ATLS,* advanced trauma life support

**Table 2:** Most-cited articles from other (non-emergency medicine) journals.

Rank	First author; year	Title	Journal: impact factor	Category	Funding (if present)	Number of citations
1	Ma, OJ; 1995	Prospective Analysis of a Rapid Trauma Ultrasound Examination Performed by Emergency Physicians	*Journal of Trauma - Injury Infection and Critical Care*; 2.7	Research		193
2	Shapiro, MJ; 2004	Simulation-based Teamwork Training for Emergency Department Staff: Does It Improve Clinical Team Performance when Added to an Existing Didactic Teamwork Curriculum?	*Quality & Safety in Healthcare*; 2.2 (2012, no longer active, title changed to BMJ Quality & Safety)	Curriculum - Yes Data	External (Army Research Laboratory Contract, AHRQ grants)	170
3	Barsuk, JH; 2009	Use of Simulation-Based Education to Reduce Catheter-Related Bloodstream Infections	*Archives of Internal Medicine*; 17.3	Curriculum - Yes Data	Combined (Excellence in Academic Medicine Act)	164
4	Stiell, I; 1995	Multicenter Trial to Introduce the Ottawa Ankle Rules for use of Radiography in Acute Ankle Injuries	*British Medical Journal*; 17.4	Curriculum - Yes Data	External (Institute for Clinical Evaluative Sciences)	147
5	Weissman, JS; 2005	Resident Physicians’ Preparedness to Provide Cross-Cultural Care	*Journal of the American Medical Association*; 35.3	Research	External (The California Endowment, The Commonwealth Fund)	123
6	Papp, KK; 2004	The Effects of Sleep Loss and Fatigue on Resident-physicians: A Multi-institutional, Mixed-method Study	*Academic Medicine*; 3.1	Research	External (National Heart, Lung and Blood Institute)	115
7	Weller, JM; 2004	Simulation in Undergraduate Medical Education: Bridging the Gap between Theory and Practice	*Medical Education*; 3.2	Curriculum - Yes Data		83
8	Larsen, DP; 2009	Repeated Testing Improves Long-term Retention Relative to Repeated Study: A Randomized Controlled Trial	*Medical Education*; 3.2	Curriculum - Yes Data	Internal	78
9	Wright, RJ; 1997	Response to Battered Mothers in the Pediatric Emergency Department: A Call for an Interdisciplinary Approach to Family Violence	*Pediatrics*; 5.5	Research	Combined (Centers for Disease Control and Prevention, NIH training grant)	62
10	Kennedy, TJT; 2007	Clinical Oversight: Conceptualizing the Relationship Between Supervision and Safety	*Journal of General Internal Medicine*; 3.4	Article/Review	External (Canadian Institutes of Health Research)	60
11	Bond, WF; 2004	Using Simulation to Instruct Emergency Medicine Residents in Cognitive Forcing Strategies	*Academic Medicine*; 3.1	Research	External (Leonard Parker Pool Healthcare Trust)	56
12	Wallin, CJ; 2007	Target-focused Medical Emergency Team Training using a Human Patient Simulator: Effects on Behaviour and Attitude	*Medical Education*; 3.2	Curriculum - Yes Data	External (Wallenberg Global Learning Network)	54
13	Baraff, LJ; 1991	Management of the Febrile Child - A Survey of Pediatric and Emergency-Medicine Residency Directors	*Pediatric Infectious Disease Journals*; 5.5	Research		52
14	Isaacson, JH; 2000	A National Survey of Training in Substance use Disorders in Residency Programs	*Journal of Studies on Alcohol*; 2.8	Research		48
15	Thomas, EJ; 2010	Team Training in the Neonatal Resuscitation Program for Interns: Teamwork and Quality of Resuscitations	*Pediatrics*; 5.5	Research	Combined (NIH)	47
16	Vaidya, NA; 2004	Relationship between Specialty Choice and Medical Student Temperament and Character Assessed with Cloninger Inventory	*Teaching and Learning in Medicine*; 0.7	Research		46
17	Baernstein, A; 2003	Promoting Reflection on Professionalism: A Comparison Trial of Educational Interventions for Medical Students	*Academic Medicine*; 3.1	Research		45
18	Kennedy, TJT; 2009	Preserving Professional Credibility: Grounded Theory Study of Medical Trainees’ Requests for Clinical Support	*British Medical Journal*; 17.4	Research	External (Canadian Institute for Health Research)	45
19	Hobgood, C; 2005	The Influence of the Causes and Contexts of Medical Errors on Emergency Medicine Residents’ Responses to their Errors: An Exploration	*Academic Medicine*; 3.1	Research		41
20	Gogalniceanu, P; 2010	Is Basic Emergency Ultrasound Training Feasible as Part of Standard Undergraduate Medical Education?	*Journal of Surgical Education*; 1.38	Curriculum - Yes Data	External (Siemens Ultrasound)	37
21	Harvey, A; 2010	Threat and Challenge: Cognitive Appraisal and Stress Responses in Simulated Trauma Resuscitations	*Medical Education*; 3.2	Research	External (Physicians Services Inc. Foundation)	37
22	Kennedy, TJT; 2009	‘It’s a Cultural Expectation...’ The Pressure on Medical Trainees to Work Independently in Clinical Practice	*Medical Education*; 3.2	Research	External (Canadian Institute for Health Research)	37
23	Revicki, DA; 1993	Organizational Characteristics, Perceived Work Stress, and Depression in Emergency-Medicine Residents	*Behavioral Medicine*; 1	Research	External (ACEP Grant)	36
24	Kennedy, TJT; 2008	Point-of-Care Assessment of Medical Trainee Competence for Independent Clinical Work	*Academic Medicine*; 3.1	Research	External (Canadian Institute for Health Research)	34
25	Kendall, JL; 2007	History of Emergency and Critical Care Ultrasound: The Evolution of a New Imaging Paradigm	*Critical Care Medicine*; 6.3	Article/Review		33

*AHRQ,* Agency for Healthcare Research and Quality; *ACEP,* American College of Emergency Physicians

**Table 3:** Papers by topic in emergency medicine journals.

Topic by list	Article/review	Curriculum	Research paper	Other	Total number (%)
Simulation	3	3	2	1	9 (36%)
Learning theory	6				6 (24%)
Ultrasound		3	2		5 (20%)
Assessment	1		2		3 (12%)
Learner retention	1				1 (4%)
Interprofessional education		1			1 (4%)
Totals	11	7	6	1	25

**Table 4:** Papers by topic in non-EM journals.

Topic by list	Article/review	Curriculum	Research paper	Other	Total number (%)
Simulation		1	5		6 (24%)
Professionalism	1	1	2		4 (16%)
Management practice		1	2		3 (12%)
Stress response		1	2		3 (12%)
Ultrasound	1		2		3 (12%)
Assessment			1		1 (4%)
Cross-cultural care		1			1 (4%)
Error in diagnosis		1			1 (4%)
Learner retention		1			1 (4%)
Specialty choice			1		1 (4%)
Supervision			1		1 (4%)
Totals	2	7	16		25
