# Supplementary material for: An Analysis of the Top-cited Articles in Emergency Medicine Education Literature
Source: West J Emerg Med. 2016 Nov 23;18(1):60–8. doi: 10.5811/westjem.2016.10.31492 (PMC5226765; doi:10.5811/westjem.2016.10.31492)
Supplement: Supplementary file 1 [file wjem-18-60-s001.docx]

Appendix 1

The journal list searched was based on journals in the Web of Science category “Emergency Medicine.” Additional journals were identified by topic searching in Web of Science as well as team member suggestions. We identified 56 journals. Following the use of inclusion and exclusion criteria, as detailed within the article, 19 journals were found to be relevant to our study. These 19 journals were Academic Emergency Medicine; American Journal of Emergency Medicine; Annals of Emergency Medicine; BMC Emergency Medicine; Canadian Journal of Emergency Medicine; Emergency Medicine Australasia; Emergency medicine Clinics of North America; Emergency Medicine Journal; European Journal of Emergency Medicine; European Journal of Trauma and Emergency Surgery; Injury; Internal and Emergency Medicine; Journal of Emergency Medicine; Journal of Trauma; Pediatric Emergency Care; Resuscitation; Scandinavian Journal of Trauma, Resuscitation and Emergency Medicine; Shock; and Western Journal of Emergency Medicine. Previous titles for the included journals were also searched. It should be noted that *BMC Emergency Medicine*, although included in PubMed, is not indexed for Web of Science. Journals excluded by the use of the above criteria included AACN Advanced Critical Care; Acute Cardiac Care; Acute Medicine; Advanced Emergency Nursing Journal; African Journal for Emergency Medicine; Air Medical Journal; Anesthesia and Resuscitation; Annals of Burns and Fire Disasters; Australasian Journal of Paramedicine; Burns: Journal of the International Society for Burn Injuries; Chinese Journal of Emergency Medicine; Clinical Pediatric Emergency Medicine; Critical Care and Resuscitation: Journal of the Australasian Academy of Critical Care Medicine; Emergency Medicine; Emergency Radiology; EMS Insider; Fire Rescue Magazine; Hong Kong Journal of Emergency Medicine; International Journal of Emergency Management; International Journal of Emergency Medicine; Journal of Acute Medicine; Journal of Burn Care and Research; Journal of Child and Adolescent Trauma; Journal of Emergencies, Trauma and Shock; Journal of Emergency Management; Journal of Emergency Medicine, Trauma and Acute care; Journal of Trauma Management and Outcomes; Open Access Emergency Medicine; Oxymag; Pediatric Critical Care Medicine; Pediatric Emergency Medicine Reports; Prehospital and Disaster Medicine; Prehospital Emergency Care; Reanimation; Traumatology; Wilderness and Environmental Medicine; and World Journal of Emergency Surgery

The keyword search for both searches included the following terms: "problem based learning,” PBL, "case based learning,” "team based learning,” "medical student*,” "medical simulation,” "multiple choice question*,” "extended matching question*,” "mini CEX,” OSCE, "formative assessment*,” "summative assessment*,” "continuing assessment*,” "online assessment*,” "self development,” "staff training,” curricul*, "program evaluation,” "self regulated learning,” "self directed learning,” professionalism, "medical ethics,” "clinical skills,” (simulat* AND patient*) or "standardized patient*,” "standardized patient*,” "medical education,” "clinical teach*,” facilitator*, tutor, tutors, learning, feedback, "role model*,” mentor*, clerk*, informatics, "e learning,” (diagnos* AND error*), "clinical reason*,” resident, residents, residency, intern, interns, internship, "house officer*,” trainee*, "house staff,” housestaff, undergraduate, graduate. Additionally, in the search involving non-emergency medicine journals, the search included “emergency medicine” and “emergency physician*,” in order to further characterize the search.
